# Development and validation of dietary and lifestyle insulinemic indices among Iranian adult population

**DOI:** 10.1186/s12986-021-00640-6

**Published:** 2022-01-10

**Authors:** Ebrahim Mokhtari, Farshad Teymoori, Hossein Farhadnejad, Parvin Mirmiran, Fereidoun Azizi

**Affiliations:** 1 Nutrition and Endocrine Research Center, Research Institute for Endocrine Sciences, Shahid Beheshti University of Medical Sciences, P.O. Box 19395-4741, Tehran, Iran; 2 Department of Clinical Nutrition and Dietetics, Faculty of Nutrition Sciences and Food Technology, National Nutrition and Food Technology Research Institute, Shahid Beheshti University of Medical Sciences, Tehran, Iran; 3 Department of Nutrition, School of Public Health, Iran University of Medical Sciences, Tehran, Iran; 4 Endocrine Research Center, Research Institute for Endocrine Sciences, Shahid Beheshti University of Medical Sciences, Tehran, Iran

**Keywords:** Hyperinsulinemia, Insulin resistance, HOMA-IR, Dietary pattern, Lifestyle, Iran

## Abstract

**Background:**

There is no study regarding developing a valid index to predict insulin-related disorders in the Iranian population based on their dietary habits and lifestyle. In the current study, we aimed to develop and validate insulinemic potential indices of diet and lifestyle in Iranian adults.

**Methods:**

In this cross-sectional study, we analysed data of 1063 men and women aged ≥ 25 years among participants of the examination three of Tehran lipid and glucose study (TLGS) (2006–2008). Dietary intakes were assessed using a valid semi-quantitative food frequency questionnaire. Dietary and lifestyle indices were developed using stepwise linear regression analysis based on dietary intakes, body mass index, and physical activity data. Fasting serum insulin concentration and homeostatic model assessment for insulin resistance (HOMA-IR) were used as biomarkers of hyperinsulinemia (HI) and insulin resistance (IR). Validation analyses were performed in examination four of TLGS.

**Results:**

We developed four indices related to insulin homeostasis, including the dietary index for HI (DIH), the dietary index for IR (DIR), the lifestyle index for HI (LIH), and the lifestyle index for IR (LIR). Based on multivariable-adjusted models, the relative values of the biomarker in subjects in the highest quartile of indices were 45% for LIH (95% CI 1.36–1.55, P_trend_ < 0.001), 28% for DIR (95% CI 1.13–1.42, P_trend_ = 0.019), and 51% for LIR (95% CI 1.41–1.61, P_trend_ < 0.001), higher than those in the reference quartile, respectively.

**Conclusion:**

We designed and validated indices to determine the insulin potential of diet and lifestyle for the Iranian population, according to Iran’s demographic and dietary intake characteristics.

**Supplementary Information:**

The online version contains supplementary material available at 10.1186/s12986-021-00640-6.

## Background

Insulin is one of the most important hormones maintaining metabolic homeostasis. Insulin affects different tissues in the body, including adipose tissue, liver, muscle, brain, bone [1], kidneys [2], and vascular system [3, 4]. Insulin-related disorders, including hyperinsulinemia (HI) and insulin resistance (IR), has been known as an early indicator and central place in metabolic dysfunction [5, 6]. If these initial disorders are left uncontrolled, they will contribute to the majority of chronic diseases such as type 2 diabetes (T2D), metabolic syndrome (MetS) [7].

Information on IR and HI statistics worldwide and in Iran is limited; however, considering the prevalence of T2D and MetS, as the two diseases most closely related to IR and HI, clarifies the current situation. According to the latest estimates, the prevalence of MetS is about 14–32% worldwide [8] and 30.8% in Iran [8]. The prevalence of diabetes in Iran in 2021 was 9.1% among adults, and it is projected to reach 10.6% by 2045. It is also estimated that the proportion of undiagnosed T2D in the Iranian adult population is about 36% [9]. This high rate of chronic diseases related to IR and HI globally, especially in Iran, requires special attention to improve the prevention and control of these disorders in the early stages. Lifestyle is the most important modifiable risk factor affecting insulin-related disorders development [10].

Diet is well-established to be an essential part of lifestyle [10]. Although specific dietary factors have been shown to affect insulin secretion and action [11–13], dietary patterns or dietary indices, including several dietary factors and cover the complex interactions between nutrients and foods, can provide a better estimate about the relationship between diet and risk of chronic disease [14, 15]. Other lifestyle factors associated with HI and IR include body mass index (BMI) and physical activity (PA) [16–18]. Given that each of these lifestyle factors is a great predictor of metabolic disorders per se, combining diet with them is likely to be more predictive of HI and IR risk than these factors alone.

Recently, Tabung et al. has developed four indices to assess the insulin potential of diet and lifestyle, including empirical dietary and lifestyle indices for HI and IR [19]. To create these indices, the concentration of connecting peptide (C-Peptide) and triglycerides (TGs) / high-density lipoprotein cholesterol (HDL-C) ratio were used as biomarkers for predicting HI and IR respectively. However, this study had some limitations. Questionnaires for medical history information were self-administered, reducing reporting accuracy [20, 21]. The study mentioned above also used blood data of participants in the Nurses’ Health Study (NHS), all of whom are women and highly educated; so, the generalizability of its results to the general population may be limited. Latterly two insulinemic indices were proposed by Mazidi et al. [22]. Similar to Tabung et al., Mazidi et al. have used C-Peptide and TG/HDL-C as biomarkers to develop the indices; however, they derived nutrient patterns, which are limited in covering interactions between different food components, such as those observed in the dietary pattern. Also, in Mazidi et al. study, the dietary intakes have assessed using a single 24-h recall, which may not be appropriate for determining the usual diet or irregularly consumed foods.

Based on the available literature on this topic, there is no study on developing a valid index to predict insulin-related disorders in the Iranian population based on their dietary habits, patterns, and lifestyle. Therefore, this study aims to develop and validate insulin potential indices of diet and lifestyle in the Iranian population.

## Methods

### Study participants

We performed the current study within the Tehran Lipid and Glucose Study (TLGS), an ongoing population-based prospective study started in 1999, which was implemented to determine the risk factors for non-communicable diseases (NCDs) among a representative urban population in Tehran, including 15,005 participants aged ≥ 3 years. The TLGS data have been collected at three-year intervals; details about TLGS have been reported elsewhere [23].

In the third examination of the TLGS (2006–2008), of 12,523 participants, 3462 were randomly selected for dietary assessment. For this study, 1286 men and women aged ≥ 25 years were selected with complete data on fasting blood sugar (FBS) and fasting serum insulin concentration. We excluded individuals based on at least one of the following criteria: history of myocardial infarction, cerebrovascular accident or cancer (n = 18), diabetic patients (n = 105), reported daily energy intakes less than 800 or more than 4200 kcal/d in men and out of the range 500–3500 kcal/d in women (n = 80), and pregnancy or lactation (n = 23); it is noticeable that some individuals fell into more than one exclusion category. Ultimately, 1063 participants remained for the analysis. For validation analysis in the fourth examination of the TLGS (2009–11), with applying the exclusion criteria mentioned above, 758 participants remained for the final analysis.

### Dietary intake assessment

We use a valid and reliable semi-quantitative 168-item food frequency questionnaire (FFQ) to assess dietary intakes at baseline. The reproducibility and validity of the FFQ have been assessed previously [24, 25]. The consumption frequency of each food item was collected during the previous year, as daily, weekly, or monthly, in a face-to-face interview by trained and skilled dieticians. Portion sizes of consumed foods were reported in household measures and then transformed into grams scale. The energy contents of consumed foods were calculated using the United States Department of Agriculture (USDA) food composition table (FCT). Furthermore, the Iranian FCT was used to analysis of the local food items that were not available in USDA FCT.

### Physical activity assessment

The modifiable activity questionnaire (MAQ) was used to assess individuals’ physical activity levels. This questionnaire has previously been modified and validated for the Iranian adult population [26]. Participants were requested to report the frequency and time spent on activities in four intensity levels, light, moderate, heavy, and very heavy, during the previous year, according to a list of daily life common activities. Ultimately, physical activity levels were expressed as metabolic equivalent hours per week (MET-h/wk.).

### Demographic and lifestyle-related measurements

Using a pretested questionnaire, trained interviewers collected information on age, sex, medical history, medication use, occupation status, and smoking. The participant’s body weight was measured to the nearest 0.1 kg using digital scales (model 707, Seca, Hamburg, Germany) while lightly clothed and barefoot. Subjects’ height was measured to the nearest 0.5 cm using a stadiometer (model 208 Portable Body Meter Measuring Device; Seca) while subjects were standing without shoes. BMI was computed as weight (kilograms) divided by height (meters squared). Waist circumference (WC) was measured to the nearest 0.1 cm using a non-elastic tape meter, between the lowest chest ribs and the iliac crest at the umbilicus level, over light clothing, without any pressure on the body skin.

### Biochemical measurements

Blood samples were taken and transferred into vacutainer tubes between 7:00 and 9:00 a.m, after a 12–14-h overnight fast, while subjects were in a sitting position. Blood samples were centrifuged within 30 to 45 min of collection. All biochemical analyses were performed using a Selectra 2 auto-analyzer at the TLGS research laboratory on the day of blood collection. FBS was measured using an enzymatic colorimetric method with glucose oxidase. Inter- and intra-assay CVs were both 2.2% for FBS [23]. TGs levels were measured using the enzymatic colorimetric method with glycerol phosphate oxidase. Inter- and intra-assay CVs for TGs were 0.6 and 1.6%, respectively. Serum HDL-C was measured after precipitation of the apolipoprotein B-containing lipoproteins with phosphotungstic acid. Fasting Insulin was measured via electrochemiluminescence immunoassay (ECLIA), using Roche Diagnostics kits and Roche/Hitachi Cobas e-411 analyzer (Gmbh, manhim, Germany). Inter- and intra-assay coefficient variations for insulin were 1.2 and 3.5, respectively.

### Definitions

**HOMA-IR = **FBS (mmol/L) × serum insulin (μU/mL) / 22.5

**Visceral adiposity index (VAI):** this index was calculated for men and women as below:

- **Males:** VAI = (WC (cm) / (39.68 + (1.88 × BMI (kg/m^2^))) × (TG (mmol/l) / 1.03) × (1.31 / HDL (mmol/l))
- **Females:** VAI = (WC (cm) / (39.58 + (1.89 × BMI (kg/m^2^))) × (TG (mmol/l) / 0.81) × (1.52 / HDL (mmol/l))

**Waist residual BMI:** we regressed WC on BMI to obtain BMI-independent waist circumference values, calculated as differences between each individual’s WC and the WC predicted by BMI.

### Development of indices

In the first stage, all food groups and subgroups that their association with the risk of IR, HI, T2D, and MetS have been considered in previous studies (especially based on studies conducted on the Iranian population) were extracted. Finally, 38 food groups were considered for development analysis, including red meat, processed meat, poultry, fish, egg, organ meats, pizza, broth, low-fat dairy, high-fat dairy, doogh, sweetened dairy, refined grain, whole grain, legumes, nuts, dried fruits, fruit juices, other fruits, lemon juices, starchy vegetables, tomato, pickles, leafy vegetables, garlic and onion, other vegetables, fried potato, animal fat, liquid oils, butter, mayonnaise, olives, sweet snacks, artificial beverages, tea, salt, coffee, herbs. The intakes of these food groups or food items were calculated as daily servings per 1000 kcal energy intake.

Stepwise linear regression analysis was conducted to derive dietary patterns associated with HI and IR, using fasting insulin concentration and HOMA-IR index (log-transformed), respectively, as the response variables and the 38 food items mentioned earlier as the predictor. The significance level was considered at *P* = 0.1 for entry into and retention in the final model. To develop lifestyle indices, BMI and PA were also added to the final model. Ultimately the linear β-regression coefficients (as the weights) and the coefficient of determination (R^2^), obtained in the final model of stepwise regression analysis, were reported for each food group.

### Components derived for each index

The dietary index for HI (DIH), the dietary index for IR (DIR), were developed based on different food items. The DIH had ten components, including refined grains, pickles, doogh (a beverage made with yogurt), sweetened beverages, fish, and lemon juice which directly related to the serum insulin concentration, and broth, red meat, high-fat dairy, starchy vegetables, which inversely related to this biomarker. The DIR was composed based on twelve food items, including six items, which had a direct relationship with the HOMA-IR index: pickles, refined grains, doogh, lemon juice, sweetened beverages, and fish, as well as six items, which had an inverse relationship with this biomarker: starchy vegetables, snacks, low-fat dairy, broth, red meat, high-fat dairy. The items of each food group are defined in detail in Additional file 1: Table S1.

The BMI, PA, and food items were considered for developing the lifestyle indices, including the lifestyle index for HI (LIH), and the lifestyle index for IR (LIR). The LIH had eight components, including BMI, refined grains, doogh, and fish (as positive components associated directly with serum insulin concentration), PA, low-fat dairy, high-fat dairy, and starchy vegetables (with inverse relationship with the serum insulin concentration). The LIR was created according to seven factors, including BMI, refined grains, and doogh, directly related to the HOMA-IR index, low-fat dairy, PA, starchy vegetables, and high-fat dairy that inversely related with HOMA-IR index.

### Calculations of indices

Weighted indices were calculated as follows: Each dietary item was converted into serving size per 1000 kcal of energy intake. Then each component, including dietary items, BMI, and physical activity (MET.h/wk.) multiplied by their weights (β coefficient) reported in Table 1, and values for all components were summed as the final score. Food groups with their items used in developed indices were defined in Additional file 1: Table S1. We also calculated unweighted forms of indices (supplementary file).

**Table 1:** Dietary and lifestyle components of the developed indices; the Third examination of Tehran Lipid and Glucose Study (2006–2008)

DIH			DIR			LIH			LIR		
Components	Weight^†^	R^2 ‡^	Components	Weight^†^	R^2 ‡^	Components	Weight^†^	R^2 ‡^	Components	Weight^†^	R^2 ‡^
*Directly related food groups*			*Directly related food groups*			*Directly related food groups*			*Directly related food groups*		
Refined grains	0.105	0.010	Pickles	0.085	0.008	BMI (kg/m^2^)	0.482	0.229	BMI (kg/m^2^)	0.487	0.232
Pickles	0.089	0.007	Refined grains	0.079	0.008	Refined grains	0.091	0.013	Refined grain	0.077	0.011
Doogh	0.082	0.006	Doogh	0.066	0.003	Doogh	0.076	0.005	Doogh	0.061	0.004
Sweetened beverages	0.062	0.005	Lemon juice	0.063	0.003	Fish	0.044	0.003			
Fish	0.061	0.003	Sweetened beverages	0.059	0.005						
Lemon juice	0.061	0.003	Fish	0.056	0.003						
*Inversely related food groups*			*Inversely related food groups*			*Inversely related food groups*			*Inversely related food groups*		
Broth	− 0.059	0.004	Starchy vegetables	− 0.054	0.003	Low fat dairy	− 0.051	0.002	Low fat dairy	− 0.063	0.003
Red meat	− 0.059	0.004	Snacks	− 0.055	0.003	Physical activity (MET-h/wk.)	− 0.064	0.004	Physical activity (MET-h/wk.)	− 0.064	0.004
High fat dairy	− 0.063	0.003	Low fat dairy	− 0.061	0.002	High fat dairy	− 0.067	0.004	Starchy vegetables	− 0.065	0.004
Starchy vegetables	− 0.068	0.004	Broth	− 0.061	0.004	Starchy vegetables	− 0.084	0.006	High fat dairy	− 0.069	0.003
			Red meat	− 0.067	0.004						
			High fat dairy	− 0.073	0.003						

The items of each food group are defined in detail in Additional file 1: Table S1

^†^β-regression coefficients were obtained from the last step of stepwise linear regression models, indicating the contribution of each component to the total index score

^‡^The coefficient of determination shows the explained variance in biomarkers by each component

### Validation analysis

To evaluate the validity of the indices for predicting the fasting serum insulin and the HOMA-IR levels based on data from the third examination of the TLGS, the developed indices were categorized into quartiles. Concentrations of the biomarkers were back-transformed to their original units. The mean and 95% confidence intervals (95% CI) of serum insulin concentration and HOMA-IR value in the first quartile of each index were assigned as the reference value. The relative values (RVs) of other quartiles were computed by dividing each quartile value by the reference value. Then the adjusted RV and 95% CI of serum insulin and HOMA-IR across quartiles of the weighted insulin-related indices were calculated using univariate linear regression analysis in two adjusted models for potential confounding variables: (1) adjusted for age and sex, and (2) adjusted for all confounding variables, including age, sex, energy intake, smoking, occupation status, medication use, as well as PA and VAI, TGs to HDL-C ratio, and waist residual BMI.

The multivariable linear regression analysis was used to compute the trend of changes in the RVs of fasting serum insulin concentration and the HOMA-IR across quartiles of each weighted index. These analyses were repeated in the next examination of the TLGS (examination 4) to confirm the validity of the weighted DIH, DIR, LIH, and LIR in an independent sample size.

### Sensitivity analysis

To evaluate the sensitivity of the developed indices, we derived three subgroups with (1) excluding individuals with a family history of T2D, (2) excluding smokers, and (3) including T2D patients in the baseline analysis. The RVs and 95% CI of serum insulin concentration and HOMA-IR were determined for each quartile of related indices, based on two above-mentioned adjusted models using univariate linear regression analysis.

### Stratified analysis

Additionally, we performed a stratified analysis to assess the distribution of the mean fasting serum insulin concentrations across quartiles of DIH and the mean of HOMA-IR across quartiles of DIR by creating two joint categories of BMI and PA as follows: active obese individuals (BMI ≥ 25 kg/m^2^ and PA ≥ median PA), and non-active obese individuals (BMI ≥ 25 kg/m^2^ and PA < median PA).

### Statistical analysis

Based on rules of thumb for determining sample size firstly proposed by Roscoe in 1975 that says in multivariate research (including multiple regression analyses), the sample size should be several times (preferably ten times or more) larger than the number of variables in the study [27, 28]. The minimum sample size for development of DIR and DIH (38 variables) and LIR and LIH (40 variables) were 380 and 400 participants, respectively. However, we developed these indices among 1063 participants in the third examination of TLGS. Validation study and other regression analysis were also conducted among sample sizes larger than estimated above.

All analyzes were performed using SPSS software version 20. The normality of the data was assessed using the Kolmogorov–Smirnov test and histogram charts. Stepwise-linear regression was used to develop insulin-related indices, including DIH, DIR, LIH, and LIR. Data are presented as the mean ± SD or the median (interquartile range) for continuous variables and percentages or numbers (percentages) for categorical variables across quartiles of DIH and DIR. Chi-square and linear regression were used to test the trend of qualitative and quantitative variables across these indices.

We computed the Pearson and Spearman correlation coefficients to evaluate the correlation between calculated indices including DIH, DIR, LIH, LIR, and also an unweighted form of these indices with and serum insulin levels and HOMA-IR in the third and fourth examination of TLGS. Furthermore, the correlation of the indices mentioned above with similar indices developed in previous studies, including insulin index, insulin load, the empirical dietary index for HI, the empirical dietary index for IR, the empirical lifestyle indices for HI, and the empirical lifestyle indices for IR, was determined.

Validation, sensitivity, and subgroup analyses were conducted using univariate linear regression analysis adjusted for potential confounders and computed the RVs and 95% CI of serum insulin concentration and HOMA-IR values across quartiles of weighted insulin indices. Then we calculated the P for trend between these RVs across quartiles of each insulin indices using multivariable linear regression. All regression analyses were adjusted for age, sex, energy intake, smoking, occupation status, medication use, PA and VAI (only for DIH and DIR), TGs to HDL-C ratio, and waist residual BMI (only for LIH and LIR). *P* values < 0.05 were considered as statistically significant.

We also calculated the power of the study for multiple linear regression using G-power software version 3.1.9.4 by considering a two-tailed test at a 5% level of significance, sample size = 1063, 38 predictors for DIR and DIH, 40 predictors for LIR and LIH, and R^2^ of final model equal to 0.049, 0.049, 0.261, 0.265 for DIR, DIH, LIR, and LIH, respectively. We observed maximum power for all indices.

## Results

Components of the four insulinemic dietary and lifestyle indices, including DIH, DIR, LIH, and LIR, with their specific weights and R^2^ values, were reported in Table 1. Based on the relationship of components with serum insulin and HOMA-IR levels, as response biomarkers for each index, the components were divided into two categories, including (1) food groups that directly related to mentioned biomarkers and (2) food groups that showed an inverse relationship to these biomarkers. The first group components directly related to serum insulin and HOMA-IR levels were given positive weights, while components with inverse relationship with the biomarkers were assigned negative weights. Detailed information about food groups with their items used in developed indices were provided in Additional file 1: Table S1.

Table 2 presents the participants’ baseline characteristics across quartiles of the DIH and DIR. Serum insulin, HOMA-IR, WC, TGs, TG to HDL-C ratio, VAI, and percentage of men were significantly increased across quartiles of DIH and DIR (P for trend =  < 0.001). In contrast, participants’ age and HDL-C concentration were decreased across quartiles of DIH and DIR (P for trend =  < 0.05).

**Table 2:** Participant characteristics across quartiles of the dietary indices of HI and IR; the Third examination of Tehran Lipid and Glucose Study (2006–2008)

	Dietary index for hyperinsulinemia				P_trend_	Dietary index for insulin resistance				P_trend_
	Q1 (n = 266)	Q2 (n = 265)	Q3 (n = 266)	Q4 (n = 266)		Q1 (n = 266)	Q2 (n = 266)	Q3 (n = 266)	Q4 (n = 265)	
Age, y	44.4 ± 13.2	43.2 ± 11.8	42.2 ± 11.4	42.2 ± 12.7	0.031	45.2 ± 13.2	42.7 ± 11.7	41.8 ± 11.5	42.3 ± 12.5	0.006
Male, (%)	38.0	40.0	45.5	55.6	< 0.001	42.5	41.4	40.6	54.7	0.003
Current smokers, (%)	13.2	10.6	13.2	16.9	0.201	16.5	9.4	13.2	14.7	0.087
Physical activity (MET-h/wk.)	27.8 (13.9–55.6)	23.6 (10.1–51.2)	25.9 (8.4–62.5)	27.8 (11.1–52.5)	0.490	27.8 (13.1–55.4)	20.8 (9.1–48.2)	27.8 (11.4–63.4)	27.3 (10.9–52.1)	0.481
Occupation status (employed), n (%)	227 (85.3)	233 (87.9)	236 (88.7)	236 (88.7)	0.592	224 (84.2)	238 (89.5)	236 (88.7)	234 (88.3)	0.231
Medication drugs use, n (%)	18 (6.8)	17 (6.4)	15 (5.6)	14 (5.3)	0.879	19 (7.1)	17 (6.4)	12 (4.5)	16 (6.0)	0.617
Family history of diabetes (%)	21.8	18.1	15.4	18.8	0.293	18.8	19.9	16.2	19.2	0.675
BMI, kg/m2	27.1 ± 4.7	27.7 ± 5.0	27.5 ± 5.1	27.3 ± 4.8	0.848	27.0 ± 4.6	27.6 ± 4.8	27.4 ± 5.1	27.8 ± 5.1	0.090
Waist, cm	89.1 ± 12.2	91.0 ± 12.8	90.8 ± 12.6	92.2 ± 12.4	0.007	89.3 ± 12.1	90.8 ± 12.2	89.9 ± 12.6	92.9 ± 13.0	0.003
Fasting blood sugar (mg/dl)	4.84 ± 0.48	4.83 ± 0.47	4.86 ± 0.54	4.86 ± 0.49	0.505	4.83 ± 0.48	4.81 ± 0.47	4.84 ± 0.50	4.89 ± 0.51	0.138
Fasting serum insulin (mU/mL)	7.9 ± 4.0	8.6 ± 4.2	9.3 ± 5.3	9.9 ± 6.1	< 0.001	7.8 ± 4.0	8.8 ± 4.3	9.1 ± 4.7	10.2 ± 6.49	< 0.001
HOMA-IR	1.73 ± 0.99	1.86 ± 0.97	2.05 ± 1.27	2.17 ± 1.45	< 0.001	1.70 ± 0.94	1.90 ± 1.03	1.97 ± 1.09	2.25 ± 1.57	< 0.001
Triglycerides(mmol/l)	1.54 ± 0.85	1.63 ± 0.91	1.54 ± 0.80	1.84 ± 1.20	0.001	1.52 ± 0.82	1.63 ± 0.93	1.57 ± 0.80	1.83 ± 1.19	< 0.001
High-density lipoprotein (mmol/l)	1.12 ± 0.25	1.09 ± 0.27	1.07 ± 0.24	1.05 ± 0.24	< 0.001	1.11 ± 0.26	1.09 ± 0.26	1.08 ± 0.26	1.05 ± 0.24	0.004
TG: HDL	1.55 ± 1.19	1.68 ± 1.19	1.60 ± 1.10	1.95 ± 1.58	0.001	1.55 ± 1.17	1.68 ± 1.24	1.60 ± 1.05	1.95 ± 1.60	0.001
Visceral adiposity index	2.50 ± 1.94	2.70 ± 1.90	2.49 ± 1.64	3.01 ± 2.43	0.006	2.46 ± 1.90	2.66 ± 1.91	2.56 ± 1.64	3.03 ± 2.45	0.002
Energy intake (Kcal)	2290 ± 654	2221 ± 655	2139 ± 673	2225 ± 737	0.229	2281 ± 687	2232 ± 660	2147 ± 629	2215 ± 744	0.176

Data are presented as the mean ± SD or as the median (IQR) for continuous variables and as percentages or numbers and percentages for categorical variables

Chi-square and linear regression were used to test the trend of qualitative and quantitative variables across quartiles of developed insulinemic indices

Pearson and Spearman’s correlations coefficients of the insulinemic dietary and lifestyle indices with serum insulin and HOMA-IR based on examinations 3 and 4 of TLGS were shown in Table 3. In examination 3 (baseline), a significant moderate correlation was observed between all developed indices (DIH, DIR, LIH, and LIR) and serum insulin and HOMA-IR levels. In contrast, in examination 4, this significant correlation was only observed for LIH with serum insulin and LIR with HOMA-IR. In Table 3, we also reported the results on the correlation of previously designed similar insulin-related indices, including insulin index, insulin load, empirical dietary indices for HI and IR, empirical lifestyle indices for HI and IR with serum insulin levels, and HOMA-IR. Although the Spearman correlation coefficients showed that three of four above mentioned indices, including empirical lifestyle index for HI, insulin index, and insulin load, were significantly correlated with serum insulin in both examinations, Pearson correlation was significant only for the including empirical lifestyle index for HI score with serum insulin. Furthermore, a weaker correlation was found between HOMA-IR and empirical dietary index for insulin resistance in examination 3 and HOMA-IR and empirical lifestyle index for insulin resistance in examinations 3 and 4.

**Table 3:** Pearson and Spearman’s correlations coefficients between the insulinemic dietary and lifestyle indices with insulin and HOMA-IR in the two examinations of TLGS

	Pearson correlation coefficients		Spearman correlation coefficients	
	TLGS. Examination 3	TLGS. Examination 4	TLGS. Examination 3	TLGS. Examination 4
***Dietary and lifestyle indices for hyperinsulinemia***	Serum insulin			
Serum insulin	1	1	1	1
DIH	0.135**	0.003	0.124**	0.011
LIH	0.306**	0.206**	0.285**	0.115**
Unweighted DIH	0.225**	0.042	0.176**	0.024
Unweighted LIH	0.302**	0.188**	0.246**	0.101**
Empirical dietary index for HI	0.025	− 0.032	0.020	− 0.016
Empirical lifestyle index for HI	0.390**	0.316**	0.313**	0.239**
Insulin index	0.027	0.058	0.091**	0.130**
Insulin load	− 0.005	0.062	0.066^*^	0.096**
***Dietary and lifestyle indices for insulin resistance***	HOMA-IR			
HOMA-IR	1	1	1	1
DIR	0.148**	0.060	0.107**	0.063
LIR	0.306**	0.208**	0.292**	0.224**
Unweighted DIR	0.224**	0.050	0.131**	0.003
Unweighted LIR	0.300*	0.220**	0.198**	0.167**
Empirical dietary index for IR	0.112**	0.042	0.076^*^	0.032
Empirical lifestyle index for IR	0.108**	0.072*	0.060	0.085^*^
Insulin index	0.029	0.069	0.010	0.040
Insulin load	0.003	0.066	− 0.020	0.034

^**^Correlation is significant at the 0.01 level (2-tailed)

^*^Correlation is significant at the 0.05 level (2-tailed)

The Relative values and 95% CIs of serum insulin concentration and HOMA-IR across quartiles of the DIH, DIR, LIH, and LIR based on the two examinations of TLGS (3 and 4) were shown in Table 4. RVs of serum insulin concentration and HOMA-IR level followed statistically significant upward trends across quartiles of most indices in both examinations. However, a significant trend for biomarkers RVs across DIH quartiles was observed only in examination 3.

**Table 4:** Relative values and 95% confidence interval of serum insulin and HOMA-IR across quartiles of the dietary and lifestyle indices in the two examinations of TLGS

	Quartiles of dietary and lifestyle indices				P_trend_
	Q1, (Ref)	Q2, RV (95% CI)	Q3, RV (95% CI)	Q4, RV (95% CI)	
DIH					
Serum insulin (TLGS examination 3, n = 1063)					
Age and sex adjusted	1.00	1.08 (1.00–1.15)	1.17 (1.10–1.25)	1.25 (1.18–1.33)	< 0.001
Multivariable-adjusted^*^	1.00	1.07 (1.00–1.15)	1.19 (1.11–1.26)	1.22 (1.15–1.29)	< 0.001
Serum insulin (TLGS examination 4, n = 758)					
Age and sex adjusted	1.00	1.05 (0.96–1.13)	1.02 (0.94—1.11)	1.05 (0.96–1.13)	0.650
Multivariable-adjusted^*^	1.00	1.05 (0.97–1.13)	1.04 (0.96–1.12)	1.04 (0.95–1.11)	0.815
LIH					
Serum insulin (TLGS examination 3, n = 1063)					
Age and sex adjusted	1.00	0.97 (0.89–1.04)	1.21 (1.13–1.28)	1.62 (1.54–1.70)	< 0.001
Multivariable-adjusted^†^	1.00	0.98 (0.91–1.06)	1.19 (1.11–1.26)	1.59 (1.52–1.67)	< 0.001
Serum insulin (TLGS examination 4, n = 758)					
Age and sex adjusted	1.00	1.06 (0.97–1.16)	1.20 (1.11–1.30)	1.48 (1.38–1.57)	< 0.001
Multivariable-adjusted^†^	1.00	1.07 (0.99–1.16)	1.21 (1.12–1.30)	1.45 (1.36–1.55)	< 0.001
DIR					
HOMA-IR (TLGS examination 3, n = 1063)					
Age and sex adjusted	1.00	1.11 (1.03–1.19)	1.15 (1.07–1.24)	1.32 (1.24–1.40)	< 0.001
Multivariable-adjusted^*^	1.00	1.11 (1.03–1.19)	1.17 (1.09–1.25)	1.28 (1.20–1.36)	< 0.001
HOMA-IR (TLGS examination 4, n = 758)					
Age and sex adjusted	1.00	1.10 (0.94–1.25)	1.14 (0.99–1.30)	1.29 (1.14–1.45)	0.008
Multivariable-adjusted^*^	1.00	1.11 (0.96–1.25)	1.11 (0.97–1.26)	1.28 (1.13–1.42)	0.019
LIR					
HOMA-IR (TLGS examination 3, n = 1063)					
Age and sex adjusted	1.00	0.97 (0.89–1.05)	1.22 (1.14–1.31)	1.70 (1.61–1.78)	< 0.001
Multivariable-adjusted^†^	1.00	0.99 (0.91–1.07)	1.20 (1.12–1.28)	1.67 (1.59–1.75)	< 0.001
HOMA-IR (TLGS examination 4, n = 758)					
Age and sex adjusted	1.00	1.08 (0.99–1.18)	1.21 (1.11–1.31)	1.53 (1.43–1.64)	< 0.001
Multivariable-adjusted^†^	1.00	1.09 (1.00–1.19)	1.22 (1.12–1.31)	1.51 (1.41–1.61)	< 0.001

^*^Adjusted for age, sex, physical activity, energy intake, smoking, occupation status, medication use, and VAI

^†^Adjusted for age, sex, energy intake, smoking, occupation status, medication use, TGs to HDL-c ratio, and waist residual BMI

In examination 3, participants in the highest quartile of DIH and LIH had 22% and 59% higher serum insulin concentrations than those in the first quartile (95% CI 1.15–1.29, and 1.52–1.67, P for trend < 0.001), respectively. Also, based on multivariable-adjusted models, the HOMA-IR index was 28% and 67% higher among individuals in the highest vs. lowest quartile of DIR and LIR (95% CI 1.20–1.36, and 1.59–1.75, P for trend < 0.001), respectively. In examination 4, the trend of the RV of serum insulin level across quartiles of DIH score was not significant; however, an upward trend was observed in mean serum insulin level and HOMA-IR index across quartiles of the DIR, LIH, and LIR. Based on multivariable-adjusted models, the RV of mean serum insulin has significantly increased across quartile of LIH (RV = 1.45; 95% CI 1.36–1.55, P_trend_ < 0.001). Also, in fully adjusted model, the RV of HOMA-IR index level have significantly increased across quartiles of DIR (RV = 1.28; 95% CI 1.13–1.42, P_trend_ = 0.019) and LIR (RV = 1.51; 95% CI 1.41–1.61, P_trend_ < 0.001).

**Fig. 1:**
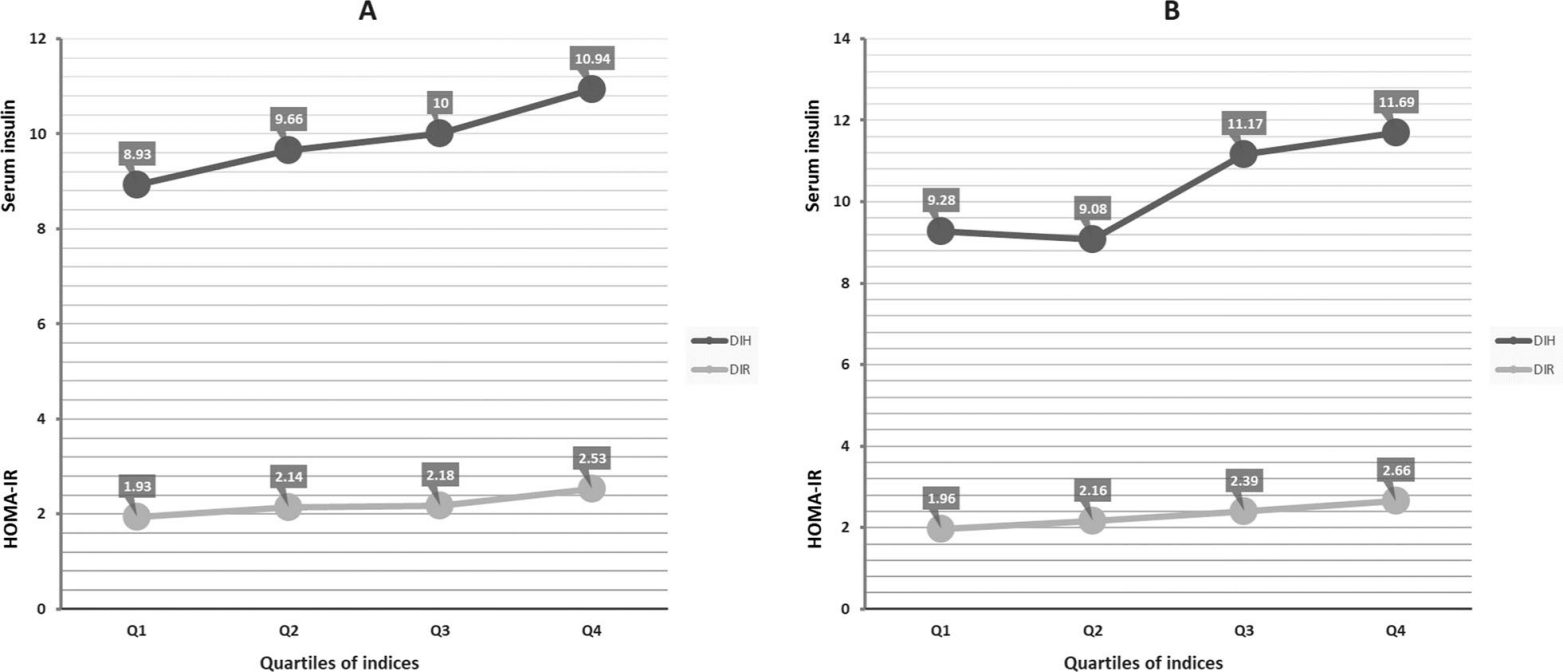
Mean values of serum insulin concentration and HOMA-IR across quartiles of DIH (black filled circle) and DIR (grey filled circle) among active obese (**A**) and non-active obese (**B**) individuals

Using the sensitivity analysis, the trend of the RV and 95% CIs of serum insulin across quartiles of DIH and LIH and the RV and 95% CIs of HOMA-IR across quartiles of DIR and LIR has been shown based on three sub-groups, including (1) excluding the smoked subjects, (2) excluding participants with a family history of T2D, and (3) including type 2 diabetic patients at baseline in Table 5. Our results showed that the RV of serum insulin concentration across quartiles has significantly increased across quartiles DIH and LIH based on age and sex adjusted and as well as multivariable-adjusted models. Also, the RV of HOMA-IR index levels has significantly increased across quartiles DIR and LIR based on both models. Furthermore, the stratified analysis showed that the mean serum insulin concentration and HOMA-IR of active-obese participants with the highest scores of DIH and DIR were 23% and 30% higher than those with the lowest scores. Similarly, non-active obese subjects in the highest quartile of DIH and DIR had 28% and 36% greater HOMA-IR values than those in the lowest quartile (Fig. 1).

**Table 5:** Relative values and 95% confidence interval of serum insulin and HOMA-IR across quartiles of the dietary and lifestyle indices after excluding the smokers, participants with a family history of diabetes, and including diabetic patients in baseline

	Quartiles of dietary and lifestyle indices				P for trend
	Q1, (Ref)	Q2, RV (95% CI)	Q3, RV (95% CI)	Q4, RV (95% CI)	
Family history DM excluded (n = 864)					
DIH					
Age and sex adjusted	1.00	1.06 (0.97–1.15)	1.20 (1.11–1.29)	1.29 (1.20–1.38)	< 0.001
Multivariable-adjusted^*^	1.00	1.05 (0.96–1.13)	1.23 (1.14–1.31)	1.25 (1.16–1.33)	< 0.001
LIH					
Age and sex adjusted	1.00	1.00 (0.91–1.09)	1.22 (1.13–1.31)	1.71 (1.62–1.79)	< 0.001
Multivariable-adjusted^†^	1.00	1.02 (0.94–1.11)	1.20 (1.11–1.29)	1.68 (1.59–1.76)	< 0.001
DIR					
Age and sex adjusted	1.00	1.12 (1.03–1.22)	1.23 (1.13–1.33)	1.38 (1.28–1.48)	< 0.001
Multivariable-adjusted^*^	1.00	1.13 (1.03–1.22)	1.25 (1.15–1.35)	1.33 (1.24–1.43)	< 0.001
LIR					
Age and sex adjusted	1.00	1.02 (0.92–1.12)	1.24 (1.14–1.34)	1.18 (1.17–1.19)	< 0.001
Multivariable-adjusted^†^	1.00	1.04 (0.94–1.13)	1.21 (1.12–1.31)	1.75 (1.66–1.85)	< 0.001
Smokers excluded (n = 920)					
DIH					
Age and sex adjusted	1.00	1.08 (1.00–1.16)	1.18 (1.10–1.26)	1.28 (1.20–1.36)	< 0.001
Multivariable-adjusted^*^	1.00	1.08 (1.01–1.16)	1.19 (1.12–1.27)	1.25 (1.17–1.32)	< 0.001
LIH					
Age and sex adjusted	1.00	1.00 (0.91–1.08)	1.23 (1.15–1.31)	1.65 (1.56–1.73)	< 0.001
Multivariable-adjusted^†^	1.00	1.01 (0.93–1.09)	1.21 (1.13–1.29)	1.62 (1.54–1.70)	< 0.001
DIR					
Age and sex adjusted	1.00	1.11 (1.02–1.20)	1.19 (1.09–1.28)	1.35 (1.26–1.44)	< 0.001
Multivariable-adjusted^*^	1.00	1.12 (1.03–1.20)	1.19 (1.10–1.28)	1.31 (1.22–1.40)	< 0.001
LIR					
Age and sex adjusted	1.00	1.00 (0.91–1.09)	1.26 (1.17–1.35)	1.73 (1.64–1.82)	< 0.001
Multivariable-adjusted^†^	1.00	1.02 (0.93–1.10)	1.24 (1.61–1.79)	1.70 (1.61–1.79)	< 0.001
Diabetic patient included (n = 1168)					
DIH					
Age and sex adjusted	1.00	0.99 (0.92–1.07)	1.15 (1.07–1.22)	1.18 (1.10–1.26)	< 0.001
Multivariable-adjusted^*^	1.00	1.00 (0.92–1.08)	1.16 (1.08–1.23)	1.15 (1.07–1.22)	0.002
LIH					
Age and sex adjusted	1.00	0.97 (0.89–1.05)	1.19 (1.11–1.12)	1.66 (1.58–1.74)	< 0.001
Multivariable-adjusted^†^	1.00	0.98 (0.90–1.06)	1.18 (1.10–1.26)	1.62 (1.54–1.70)	< 0.001
DIR					
Age and sex adjusted	1.00	1.06 (0.94–1.19)	1.12 (0.99–1.24)	1.22 (1.10–1.35)	0.005
Multivariable-adjusted^*^	1.00	1.07 (0.95–1.18)	1.11 (0.99–1.22)	1.19 (1.07–1.30)	0.040
LIR					
Age and sex adjusted	1.00	0.95 (0.81–1.03)	1.20 (1.06–1.33)	1.80 (1.67–1.94)	< 0.001
Multivariable-adjusted^†^	1.00	0.96 (0.84–1.09)	1.19 (1.06–1.31)	1.73 (1.61–1.86)	< 0.001

^*^Adjusted for age, sex, physical activity, energy intake, smoking, occupation status, medication use, and VAI

^†^Adjusted for age, sex, energy intake, smoking, occupation status, medication use, TGs to HDL-c ratio, and waist residual BMI

## Discussion

In the present study, we developed and validated four indices, including DIH and DIR, consisting of a dietary pattern predictive of HI and IR, as well as LIH and LIR encompassing both diet and lifestyle factors that are significantly related to insulin disorders. Considering the importance of PA and obesity in HI and IR [16, 18], we performed a subgroup analysis based on the physically active obese group and physically inactive obese individuals, separately. Our analysis showed that in the physically active obese subgroup, subjects in the highest quartile of DIH and DIR compared to those in the lowest one had 22 and 31% higher serum insulin and HOMA-IR, respectively. Also, in the physically inactive obese subgroup, serum insulin concentration and HOMA-IR levels of individuals in the highest quartile of DIH and DIR were 26% and 36% higher than those in the lowest quartile, respectively. These analyses showed that individuals with higher DIH and DIR scores had higher serum insulin concentration and HOMA-IR levels, independent of BMI and PA.

In recent years, Tabung et al. [19] and Mazidi et al. [22], in two separate studies, introduced several indices related to insulin homeostasis status, using serum C-Peptide concentration and TGs to HDL-C ratio as biomarkers of HI and IR, respectively. Similar to our study, Tabung et al. derived four dietary and lifestyle indices with different food groups, BMI, and PA, which indicate the insulinemic potential of diet and lifestyle; however, Mazidi’s study used a pattern of nutrients to develop insulinemic dietary indices. Using nutrients instead of food groups in Mazidi’s study may not accurately estimate the diet-disease relationship due to ignoring the complex interactions of different substances in whole foods [29]. However, it seems that the most important concern about these indices introduced in Mazidi et al. and Tabung et al.’s studies is that they are all developed in the US population, which may limit the generalizability of their results for other populations. In this study, of all food groups that were entered in the stepwise regression analysis, only those with statistically significant (positive or negative) relationships (*P* < 0.1) with serum insulin level and HOMA-IR were included in the final patterns. In our study, intakes of red meat, broth, high- and low-fat dairy products, starchy vegetables, snacks, and higher PA level showed an inverse relationship with serum insulin level and HOMA-IR, while consumption of refined grains, sweetened beverages, pickle, doogh, lemon juice, fish and BMI directly associated with the biomarkers as mentioned earlier. Our findings are in line with the results of most of the studies that have suggested the reduced risk of HI and IR in individuals with higher intakes of starchy vegetables, low-fat dairy products, and higher PA levels, as well as lower intake of refined grains and sweetened beverages and obesity [30–35].

In Iranian food culture, fish is mainly eaten fried form, which this cooking method can increase the intake of trans fats and weaken the beneficial properties of this food item. Such an effect is also noticed in other studies [19]. In contrast, red meat is mainly eaten unprocessed form along with legumes, vegetables, which may mitigate its potentially harmful effects based on results of previous studies [36]. Based on the extracted patterns in this study, higher consumption of doogh (a beverage made from yogurt, which has relatively high salt content), lemon juice, and pickles directly correlated with serum insulin and HOMA-IR levels. Although doogh is a low-calorie beverage, its high salt content can worsen IR through several mechanisms [37]. Moreover, dietary constituents like doogh, lemon juice, and pickles are often eaten with high-carbohydrate, high-calorie meals, meaning that consuming more indirectly indicates higher energy and carbohydrate intake, which raises IR risk.

In our study, lifestyle indices (LIH and LIR) were 2 to 2.5 times more correlated with biomarker levels than dietary indices (DIH and DIR). This higher correlation indicates the higher potential of lifestyle indices to predict the risk of HI and IR. Dietary indices showed a relatively low but significant correlation with serum insulin and HOMA-IR level. The low correlation of these dietary indices with the biomarkers does not mean that the developed indices are not valid; because the serum insulin and glucose level’ specialty in healthy adults, more influenced by genetic, endogenous factors (like regulatory systems), and other lifestyle factors (such as PA and BMI) compared with a usual diet [38], so the low correlation of some nutritional factors with serum insulin and HOMA-IR level was expectable. Similar findings have been observed in other studies [19, 39], e.g., in the Fung et al. study, a dietary pattern predicting serum C-peptide showed a relatively low correlation (r = 0.23) with the levels of this biomarker; however, the association of this pattern with the risk of colon cancer was still significant [39]. Also, in the Tabung et al. study, the reported correlation coefficients between developed indices and biomarkers were close to ours, and their indices had good predictive power for the risk of HI and IR [19].

Various analyses confirmed the robustness and validity of all four developed indices in our study. In the third examination of the TLGS study (baseline), the relative serum insulin concentration and HOMA-IR levels increased across the quartiles of DIH, DIR, LIH, and LIR with a significant trend. Similarly, these significant findings were observed in the fourth examination for all indices mentioned above except DIH. In general, dietary indicators are less capable than lifestyle indicators for predicting the response biomarkers. In the validation study, the adjusted RV of fasting insulin in the highest vs. lowest quartile of DIH was 4% higher; however, it showed no significant trend. In contrast, HOMA-IR showed a significant positive trend across quartiles of DIR. Some possible reasons may explain why DIH has shown a weaker predictive ability than DIR. First, DIH covers a smaller range of dietary components than DIR, as DIR, additional to similar items with DIH, includes snacks and low-fat dairy. Second, fasting insulin concentration (as the response variable for DIH) is less affected by diet compared with HOMA-IR (as the response variable for DIR) because FBS as a component of HOMA-IR is more affected by food intakes [40]. Finally, in our study, about 97% of individuals had a positive DIH score (compared to DIR with 75% positive and 25% negative). This may have undermined the DIH’s ability to discriminate between individuals with different serum insulin levels and, consequently, its predictive ability in our study.

Furthermore, we tested the sensitivity of the indices in three different sub-groups by excluding subjects with a family history of T2D, excluding smoked participants, and including diabetic patients in the analysis. Finally, based on each sub-group analysis, the relative values of serum insulin concentration and HOMA-IR significantly increased across quartiles of all developed indices.

We examined the correlation of the developed indices in the unweighted form and the similar indices developed in previous studies with the serum insulin and HOMA-IR levels. Our results showed that even unweighted forms of DIH and DIR correlate with serum insulin concentration and HOMA-IR more than the empirical dietary index for HI, insulin index, and insulin load. Weighted DIH and DIR showed a lower correlation with biomarkers than unweighted forms. One possible reason could be that in unweighted form, all components were equally involved in the correlation between indices and biomarkers regardless of their dietary intakes, e.g., in the weighted form, the effect of some components such as pickles that were high in weight and could increase correlation was attenuated by low dietary intake, The opposite is true for sweetened beverages. Among lifestyle indices, the large difference in weight and R^2^ of the BMI compared to other items covered their overall impact and significantly affected increasing correlation. When BMI was considered with the same weight as other factors, its effect is attenuated, which reduces the correlation of index with serum insulin and HOMA-IR. Further analysis showed that the DIH, DIR, LIH, and LIR were significantly correlated (r < 0.40) with their unweighted forms (Additional file 1: Table S2, S3).

The indices introduced in the present study can be used in other populations, especially those with similar habits to the Iranian population, such as people in the Caucasus and the Middle East. The main benefit of our results is that researchers who do not have access to insulin biomarkers data can easily estimate the insulinemic potential of diet and lifestyle using only dietary and lifestyle data. More research and studies are still needed on the derived patterns and the food groups (to determine the optimal intakes). If the results obtained in this study are repeated in future studies, clinical applications for these findings can be considered. Physicians and dietitians can assess the diet and lifestyle of patients at risk of developing insulin-related disorders using the indices proposed in this study.

Our study had several strengths and limitations. This work is the first study conducted to develop and validate dietary and lifestyle indices to predict the risk of HI and IR among the Iranian population. We used serum insulin concentration as a response variable to develop DIH and LIH indices, which is a direct indicator for the diagnosis of HI. HOMA-IR index was also used to detect insulin resistance for DIR and LIR development. This index has been widely used in studies among various populations and on several outcomes due to the high correlation with the euglycemic hyperinsulinemic clamp techniques as the gold standard for diagnosing IR [41]. Our study has some limitations. First, due to the lack of another study on measuring the serum insulin concentration with an acceptable sample size in the Iranian adult population, we had to validate the developed indices using participants’ data from examination 4 of the TLGS study. However, this limitation is somewhat negligible because the validation of the indices was done in the fourth examination of the TLGS study with new entries and different sample sizes, in which their data were collected independently of the third examination (baseline). Second, despite using a valid and reliable FFQ for dietary assessment, measurement errors were inevitable in the estimates of dietary intake using FFQ. However, we entered dietary intakes as energy-adjusted into analysis to reduce the impact of measurement errors to the extent possible. Third, we have no complete data of other lifestyle factors such as income, culture, customs, etc. data to determine better the lifestyle index related to HI and IR. Fourth, although we attempted to adjust for major confounders in the present study, the confounding effect of some unknown and unmeasured residual confounders cannot be excluded. In addition, although direct measurement of PA is not common in large population studies, the use of indirect measurement for PA is another limitation that cannot be ignored completely; however, we collected the PA data using a valid questionnaire in a face-to-face interview to enhance the assessment precision.

## Conclusion

In the present study, we have designed and validated four insulinemic indices, including DIH, DIR, LIH, and LIR, to determine the insulinemic potential of diet and lifestyle in the Iranian population, according to their demographic and dietary intakes. The findings of this study can be used for research and clinical purposes. These indicators may help identify individuals, groups, and even populations at risk for diabetes, metabolic syndrome, cardiovascular disease, etc. Researchers who do not have access to insulin biomarker data and researchers in other countries, especially in Southwest Asia, which has a food culture and lifestyle close to Iran, can use these indices in their studies. The results of this study need to be confirmed by large prospective studies and randomized clinical trials.

## Supplementary Information

**Additional file 1. Table S1.** Food groups with their items used in developed indices. **Table S2.** Pearson and Spearman’s correlations coefficients between the insulinemic dietary and lifestyle patterns in the two examinations of TLGS. **Table S3.** Pearson and Spearman’s correlations coefficients between the insulinemic dietary and lifestyle patterns in the two examinations of TLGS. An alternative method for the calculation of indices. Calculation of unweighted forms of indices.

## Data Availability

The data underlying this article will be shared at reasonable request to the corresponding author.

## Abbreviations

BMI: Body mass index
CI: Confidence interval
C-Peptide: Connecting peptide
DIH: Dietary index for HI
DIR: Dietary index for IR
ECLIA: Electrochemiluminescence immunoassay
FBS: Fasting blood sugar
FCT: Food composition table
FFQ: Food Frequency Questionnaire
HDL-C: High-density lipoprotein cholesterol
HI: Hyperinsulinemia
HOMA-IR: Homeostatic model assessment for insulin resistance
IQR: Interquartile range
IR: Insulin resistance
LIH: Lifestyle index for HI
LIR: Lifestyle index for IR
MAQ: Modified Activity Questionnaire
MetS: Metabolic syndrome
NCDs: Non-communicable diseases
NHS: Nurses’ Health Study
PA: Physical activity
R^2^: Coefficient of determination
RV: Relative value
SD: Standard deviation
T2D: Type 2 diabetes
TGs: Triglycerides
TLGS: Tehran lipid and glucose study
USDA: United States Department of Agriculture
VAI: Visceral adiposity index
WC: Waist circumference
