# Supplementary material for: Development and validation of dietary and lifestyle insulinemic indices among Iranian adult population
Source: Nutr Metab (Lond). 2022 Jan 10;19:5. doi: 10.1186/s12986-021-00640-6 (PMC8751331; doi:10.1186/s12986-021-00640-6)
Supplement: Supplementary file 1 — Additional file 1. Table S1. Food groups with their items used in developed indices. Table S2. Pearson and Spearman’s correlations coefficients between the insulinemic dietary and lifestyle patterns in the two examinations of TLGS. Table S3. Pearson and Spearman’s correlations coefficients between the insulinemic dietary and lifestyle patterns in the two examinations of TLGS. An alternative method for the calculation of indices. Calculation of unweighted forms of indices. [file 12986_2021_640_MOESM1_ESM.docx]

**LIST OF SUPPLEMENTARY MATERIALS**

**Table S1.** Food groups with their items used in developed indices (serving sizes converted to grams / d).

**Table S2.** Pearson and Spearman’s correlations coefficients between the insulinemic dietary and lifestyle patterns in the two examinations of TLGS

**Table S3.** Pearson and Spearman’s correlations coefficients between the insulinemic dietary and lifestyle patterns in the two examinations of TLGS

Alternative method for calculation of indices

Calculation of unweighted form of indices

**Table S1.** Food groups with their items used in developed indices (serving sizes converted to grams / d).

| **Food groups** | **Food items** |
| --- | --- |
|  |  |
| Red meat | 30 gr beef or calf, 30 gr lamb meat, 30 gr ground beef |
| Fish | 30 gr fish (except tuna), 30 gr tuna (canned) |
| Broth | 235 gr broth |
| Low-fat dairy | 245 gr skim milk, 245 gr low-fat milk (less than 2%), 183 gr regular yogurt |
| High-fat dairy | 245 gr whole milk (greater or equal to 2%), 178 gr ooze yogurt, 183 gr full fat yogurt, 178 gr cream yogurt, 30 gr cheese, 30 gr cream cheese, 178 gr cream |
| Doogh | 490 gr doogh |
| Refined grain | 30 gr lavash bread, 30 gr baguette bread, 79 gr cooked rice, 180 gr cooked pasta, 180 gr baked vermicelli (soup noodle), 180 gr āsh noodle |
| Starchy vegetables | 173 gr potato, 150 gr corn, 122.5 gr pumpkin, 90 gr stewed pumpkin, green 80 gr peas cooked, 62.5 gr green beans cooked |
| Pickles | 122.5 gr pickles, 122.5 gr SHOOR, 90 gr pickled cucumber |
| Sweetened beverages | 120 gr canned fruits syrup, 120 gr industrial or cola beverages |
| Snacks | 15 gr sugar cube, 15 gr comfit, 15 gr sugar, 15 gr honey, 15 gr jams, 15 gr HALVA, 15 gr tahini HALVA, 30 gr cookies, 30 gr crackers (crispy biscuits), 30 gr different types of cakes, 15 gr dried sweets, 15 gr cream sweets, 15 gr GAZ, 15 gr SOHAN, 26 gr chocolate, 54 gr doughnut |
| Lemon juice | 15 gr lemon juice |

**Table S2.** Pearson and Spearman’s correlations coefficients between the insulinemic dietary and lifestyle patterns in the two examinations of TLGS

|  | **Pearson Correlation** | | | |  | **Spearman Correlation** | | | |
| --- | --- | --- | --- | --- | --- | --- | --- | --- | --- |
|  | DIH | LIH | Unweighted DIH | Unweighted LIH |  | DIH | LIH | Unweighted DIH | Unweighted LIH |
| **TLGS. Phase 3 (n=1063)** |  |  |  |  |  |  |  |  |  |
| DIH | 1 | 0.011 | 0.441^**^ | 0.486^**^ |  | 1 | 0.027 | 0.447^**^ | 0.482^**^ |
| LIH | 0.011 | 1 | 0.080^**^ | 0.400^**^ |  | 0.027 | 1 | 0.071^*^ | 0.426^**^ |
| Unweighted DIH | 0.441^**^ | 0.080^**^ | 1 | 0.588^**^ |  | 0.447^**^ | 0.071^*^ | 1 | 0.516^**^ |
| Unweighted LIH | 0.486^**^ | 0.400^**^ | 0.588^**^ | 1 |  | 0.482^**^ | 0.426^**^ | 0.516^**^ | 1 |
| EDIH | 0.004 | -0.066^*^ | 0.085^**^ | 0.041 |  | 0.020 | -0.066^*^ | 0.095^**^ | 0.067^*^ |
| ELIH | 0.003 | 0.590^**^ | 0.064^*^ | 0.360^**^ |  | 0.020 | 0.611^**^ | 0.051 | 0.355^**^ |
| Insulin index | 0.349^**^ | -0.032 | 0.106^**^ | 0.106^**^ |  | 0.297^**^ | 0.014 | 0.090^**^ | 0.085^**^ |
| Insulin Load | 0.276^**^ | -0.079^**^ | 0.058 | 0.099^**^ |  | 0.166^**^ | -0.049 | 0.032 | 0.072^*^ |
| **TLGS, Phase 4 (n=**758**)** |  |  |  |  |  |  |  |  |  |
| DIH | 1 | -0.059 | 0.442^**^ | 0.397^**^ |  | 1 | -0.020 | 0.517^**^ | 0.440^**^ |
| LIH | -0.059 | 1 | -0.009 | 0.406^**^ |  | -0.020 | 1 | -0.022 | 0.381^**^ |
| Unweighted DIH | 0.442^**^ | -0.009 | 1 | 0.586^**^ |  | 0.517^**^ | -0.022 | 1 | 0.499^**^ |
| Unweighted LIH | 0.397^**^ | 0.406^**^ | 0.586^**^ | 1 |  | 0.440^**^ | 0.381^**^ | 0.499^**^ | 1 |
| EDIH | 0.186^**^ | -0.085^*^ | 0.176^**^ | 0.099^**^ |  | 0.205^**^ | -0.058 | 0.178^**^ | 0.091^*^ |
| ELIH | 0.044 | .0586^**^ | -0.018 | 0.371^**^ |  | 0.072^*^ | 0.570^**^ | -0.024 | 0.368^**^ |
| Insulin index | 0.326^**^ | -0.066 | 0.009 | 0.027 |  | 0.156^**^ | -0.061 | -0.003 | 0.015 |
| Insulin Load | 0.236^**^ | -0.138^**^ | 0.076^*^ | 0.023 |  | 0.183^**^ | -0.144^**^ | 0.055 | 0.004 |

^**^Correlation is significant at the 0.01 level (2-tailed).

^*^Correlation is significant at the 0.05 level (2-tailed).

**Table S3.** Pearson and Spearman’s correlations coefficients between the insulinemic dietary and lifestyle patterns in the two examinations of TLGS

|  | **Pearson Correlation** | | | |  | **Spearman Correlation** | | | |
| --- | --- | --- | --- | --- | --- | --- | --- | --- | --- |
|  | DIR | LIR | Unweighted DIR | Unweighted LIR |  | DIR | LIR | Unweighted DIR | Unweighted LIR |
| **TLGS. Phase 3 (n=1063)** |  |  |  |  |  |  |  |  |  |
| DIR | 1 | 0.025 | 0.558^**^ | 0.555^**^ |  | 1 | 0.037 | 0.544^**^ | 0.520^**^ |
| LIR | 0.025 | 1 | 0.083^**^ | 0.392^**^ |  | 0.037 | 1 | 0.053 | 0.409^**^ |
| Unweighted DIR | 0.558^**^ | 0.083^**^ | 1 | 0.569^**^ |  | 0.544^**^ | 0.053 | 1 | 0.499^**^ |
| Unweighted LIR | 0.555^**^ | 0.392^**^ | 0.569^**^ | 1 |  | 0.520^**^ | 0.409^**^ | 0.499^**^ | 1 |
| EDIR | 0.558^**^ | -0.019 | 0.285^**^ | 0.317^**^ |  | 0.468^**^ | -0.003 | 0.263^**^ | 0.301^**^ |
| ELIR | 0.697^**^ | 0.027 | 0.259^**^ | 0.428^**^ |  | 0.603^**^ | 0.051 | 0.260^**^ | 0.419^**^ |
| Insulin index | 0.204^**^ | -0.034 | -0.012 | 0.128^**^ |  | 0.171^**^ | 0.009 | -0.021 | 0.108^**^ |
| Insulin Load | 0.196^**^ | -0.081^**^ | 0.016 | 0.155^**^ |  | 0.106^**^ | -0.054 | -0.006 | 0.130^**^ |
| **TLGS, Phase 4 (n=**758**)** |  |  |  |  |  |  |  |  |  |
| DIR | 1 | -0.029 | 0.534^**^ | 0.466^**^ |  | 1 | 0.006 | .550^**^ | 0.464^**^ |
| LIR | -0.029 | 1 | 0.000 | 0.429^**^ |  | 0.006 | 1 | -0.021 | 0.415^**^ |
| Unweighted DIR | 0.534^**^ | 0.000 | 1 | 0.556^**^ |  | 0.550^**^ | -0.021 | 1 | 0.482^**^ |
| Unweighted LIR | 0.466^**^ | 0.429^**^ | 0.556^**^ | 1 |  | 0.464^**^ | 0.415^**^ | 0.482^**^ | 1 |
| EDIR | 0.566^**^ | -0.047 | 0.301^**^ | 0.285^**^ |  | 0.460^**^ | -0.055 | 0.277^**^ | 0.246^**^ |
| ELIR | 0.731^**^ | -0.038 | 0.256^**^ | 0.392^**^ |  | 0.537^**^ | 0.001 | 0.247^**^ | 0.395^**^ |
| Insulin index | 0.202^**^ | -0.067 | -0.071 | 0.117^**^ |  | 0.108^**^ | -0.058 | -0.042 | 0.086^*^ |
| Insulin Load | 0.212^**^ | -0.138^**^ | 0.054 | 0.066 |  | 0.180^**^ | -0.145^**^ | 0.056 | 0.056 |

^**^Correlation is significant at the 0.01 level (2-tailed).

^*^Correlation is significant at the 0.05 level (2-tailed).

**Alternative method for** **calculation of indices:**

In addition to the method introduced in the main article, the calculation of indices can be done through an alternative method by multiplying the Z-scores of BMI, physical activity (MET.h/Wk), and dietary items (serving size in 1000 Kcal) by weights (β coefficient) reported in table 1 and then values for all components be summed as the final score. This method can reduce the magnitude of the calculated scores.

**Calculation of unweighted form of indices:**

All components (BMI, physical activity, and dietary items (serving size in 1000 Kcal)) of each index were categorized into quintiles. Subject’s quintile rankings were used to determine component scores, as participants in the lowest quintile of components with positive associations were received 1 point, and those in the highest one was received 5 points. For components with inverse associations, those in the lowest quintile were assigned 5 points, and those in the highest one was assigned 1 point. We then summed the scores of all components for each index to obtain overall score.
